# Statins in Candidemia: clinical outcomes from a matched cohort study

**DOI:** 10.1186/1471-2334-10-152

**Published:** 2010-06-04

**Authors:** Graeme N Forrest, Angela M Kopack, Eli N Perencevich

**Affiliations:** 1 Division of Infectious Diseases, Oregon Health Sciences University and Portland VA Medical Center, Portland, 3701 SW US Veterans Hospital Rd, P3-ID, Portland, OR, 97239, USA; 2 Infectious Diseases Associates, Ellicott City, MD, 21201, USA; 3 Professor of Medicine, University of Iowa Carver College of Medicine, Iowa City VA Medical Center, 200 Hawkins Dr, SE620GH, Iowa City, IA, 52242, USA

## Abstract

**Background:**

HMG CoA reductase inhibitors (statins) in patients with bacteremic sepsis have shown significant survival benefits in several studies. There is no data on the effect of statins in candidemic patients, however in-vitro models suggest that statins interfere with ergesterol formation in the wall of yeasts.

**Methods:**

This retrospective matched- cohort study from 1/2003 to 12/2006 evaluated the effects of statins on patients with candidemia within intensive care units. Statin-users had candidemia as a cause of their systemic inflammatory response and were on statins throughout their antifungal therapy, while non-statin users were matched based on age +/- 5 years and co-morbid factors. Primary analysis was 30-day survival or discharge using bivariable comparisons. Multivariable comparisons were completed using conditional logistic regression. All variables with a p-value less than 0.10 in the bivariable comparisons were considered for inclusion in the conditional logistic model.

**Results:**

There were 15 statin-users and 30 non-statin users that met inclusion criteria, all with similar demographics and co-morbid conditions except the statin group had more coronary artery disease (P < 0.01) and peripheral vascular disease (P = 0.03) and lower median APCAHE II scores (14.6 vs 17, p = 0.03). There were no differences in duration of candidemia, antifungal therapy or *Candida *species between the groups. Statins were associated with lower mortality on bivariable (OR 0.09, 95% CI 0.11-0.75, p = 0.03) and multivariable (OR 0.22, 95% CI 0.02-2.4, p = 0.21) analyses compared to controls; although, in the latter the protective effect lacked statistical signficance.

**Conclusion:**

In our small, single-center matched-cohort study, statins may provide a survival benefit in candidemia, however further studies are warranted to validate and further explore this association.

## Background

Candidemia is the fourth common cause of nosocomial blood stream infections and is associated with a significant mortality [1,2]. Delays in antifungal therapy have been associated with increased hospital costs of over $6,000 per patient and overall mortality [3,4]. The role of 3-hydroxy-3methylglutaryl-coenzyme A (HMG CoA) reductase inhibitors (statins) in improving outcomes bacteremic sepsis is currently being debated with recent papers showing significantly improved survival in patients with systemic inflammatory response syndrome (SIRS) in the intensive care unit (ICU), in patients with chronic kidney renal disease and patients with community acquired pneumonia and influenza [5-9]. One explanation of this effect is that statins in animal models have shown to reduce inflammatory markers, in particular the release of cytokines and cytotoxic effects of neutrophils [10,11]. The reduction in inflammatory cytokines has also been demonstrated in patients in a prospective randomized study comparing simvastatin to placebo where there was a significant reduction in tumor necrosis factor alpha (TNF-α) and interleukin-6 (IL-6) in the statin group [12].

Yeasts use the same HMG CoA reductase as humans, however their end-product is ergosterol rather than cholesterol [13,14]. In-vitro studies have demonstrated that simvastatin greatly inhibits the growth of *Candida *species [15,16]. We performed a review to determine if there was a clinical benefit of statin therapy throughout antifungal therapy in intensive care unit patients with confirmed candidemia.

## Methods

### Setting

This study was performed at the University of Maryland Medical Center, a tertiary care hospital with over 200 intensive care unit (ICU) beds, and the study was approved by the University of Maryland Institutional Review Board.

### Definitions

We completed a retrospective matched-cohort study performed between 1/2003 and 12/2006. Statin-users included were at least 45 years old, taking a statin (simvastatin, atorvastatin, or pravastatin) present in any medical or surgical ICU at the onset of candidemia. The exposed patients or statin-users had to remain on a statin from the onset of their candidemia to the end of appropriate antifungal therapy and have a SIRS response (based on Society of Critical Care Medicine definitions [17]). For every statin-user, there were 2 matched unexposed, non-statin users based on age within 5 years of each case, co-morbid conditions and antifungal therapy who developed candidemia within an ICU while present in any ICU.

Only the initial episode of candidemia per patient was evaluated for both groups and statin-users and non-statin users were excluded if there was positive blood cultures for any other organism within 24 hours either side of the sentinel blood culture for yeast. Statin users and non-users were also excluded if another active cause for the SIRS response was found (e.g hospital acquired pneumonia, urosepsis, *Clostridum difficile *colitis, or catheter-related bacteremia), or if they died either prior to the diagnosis of candidemia or before receiving any antifungal therapy.

### Data collection

The data collected from both groups included patient demographics, co-morbid conditions, the age adjusted Charlson Comorbidity Index (CCI) [18], APACHE II score at the time of onset of the index candidemia blood draw, the type and dose of statin used, *Candida *species, initial empiric antifungal therapy, and 30-day survival or discharge. The primary aim was to determine whether statin-users had improved survival over a similarly matched-cohort of non-statin users.

### Statistical analysis

Bivariable comparisons were completed using t-tests for normally distributed continuous variables, Wilcoxon rank sum for non-normally distributed continuous variables and the Fisher-exact test for categorical variables. Bivariable statistical comparisons presented in Table 1 were completed without considering the matching. Matched bivariable comparisons and multivariable comparisons were completed using conditional logistic regression. All variables in the bivariable comparisons were considered for inclusion in the conditional logistic model. Each individual parameter was then analyzed in the conditional model by itself to determine significant association with mortality under a matched analysis and then was added into the multivariable model and left in the model if it confounded the primary association between statin exposure and mortality with a 10% or greater change in the point estimate. The statin exposure was forced into the models, as it was the primary exposure of interest. All analyses were completed using SAS version 9.1 (Cary, NC).

**Table 1:** Demographics, co-morbid conditions and antifungal therapy of study groups.

Variable	Patients on Statins N (% or +/- SD)	Patients Not on Statins N (% or SD)	p-value†
Age (years)	65.9 (+/- 9.4)	67.7 (+/- 8.7)	0.52

Male	11 (73%)	12 (40%)	0.06

Medical ICU	9 (60%)	24 (80%)	1

Median Charlson Score (IQR)	8 (5-8)	6 (5-7)	0.15

Apache II	14.9 (+/- 3.8)	18.2 (+/- 5.0)	0.03*

AST	20 [17,140]	34 [24,86]	0.34

ALT	22 [19,135]	44 [24,113]	0.17

Albumin	2 (+/- 0.3)	2 (+/- 0.5)	0.76

Bicarbonate	23.9 (+/- 5.6)	22.9 (+/- 5.5)	0.57

Creatinine	2.8 (+/- 2.3)	2.7 (+/- 1.7)	0.80

CPK	129 [39,1502]	45 [35,163]	0.45

Hematocrit	29.8 (+/- 4.2)	29.2 (+/- 4.9)	0.66

PT	14.2 (+/- 2.4)	14.0 (+/- 2.9)	0.79

PTT	31.6 (+/- 8.6)	39.3 (+/- 16.9)	0.06

WBC	12.2 (+/- 4.5)	16.8 (+/- 8.8)	0.02*

Platelets	241 (+/- 127)	221 (+/- 130)	0.62

Heart disease	15 (100%)	16 (53%)	<0.01*

Heart failure	7 (47%)	6 (20%)	0.08

Obstructive lung disease	4 (27%)	8 (27%)	1.0

Renal insufficiency	7 (47%)	7 (23%)	0.08

Dialysis	4 (27%)	9 (30%)	1.0

Stroke	6 (40%)	7 (23%)	0.3

Cancer	2 (13%)	4 (13%)	1.0

Diabetes	10 (67%)	17 (57%)	0.75

HIV/AIDS	0	0	

Hypertension	12 (80%)	20 (67%)	0.49

Peripheral vascular disease	5 (33%)	2 (7%)	0.03*

Transplantation	1 (7%)	1 (3%)	1.0

Median duration of Candidemia (days)	2 (+/-0.6)	1 (+/-0.9)	0.2

Survival	11 (73%)	11 (37%)	0.02*

* Significant values, p < 0.05

APACHE II: Acute Physiology and Chronic Health Evaluation II, AST: aspartate transaminase, ALT: alanine transaminase, CPK: creatine phosphokinase, ICU: Intensive Care Unit, IQR; Interquartile range, PT: Prothrombin time, PTT: partial thromboplastin time, WBC; white blood cell count.

†All continue variables presented as mean, standard deviation (SD) except ALT, AST, and CPK, which were non-normally distributed and presented as median with interquartile range.

## Results

**Figure 1:**
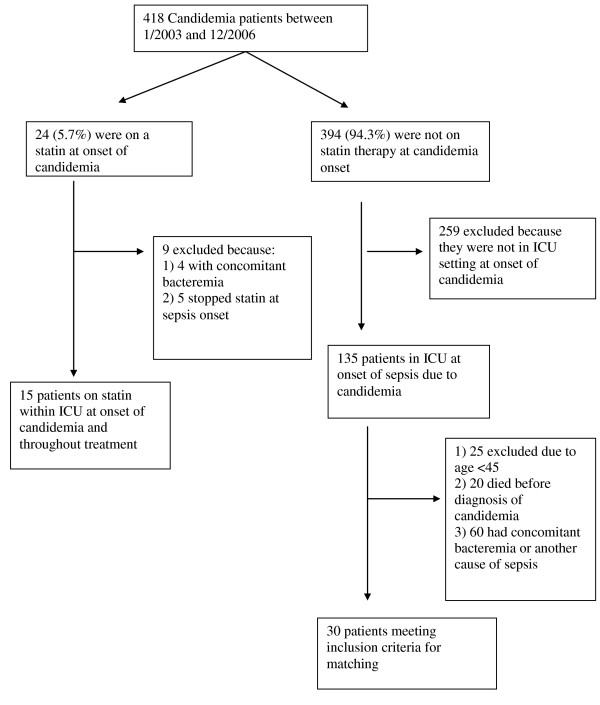
**Distribution of candidemic patients by statin and non-statin-user**. Footnote: ICU = Intensive care unit

Out of a total of 418 individual episodes of candidemia during the four-year study period, there were 24 (5.7%) patients on statin therapy. After exclusion criteria were included, there were 15 statin users and 30 non-statin users who met the full inclusion criteria. (Figure 1) The main demographic and comorbid differences (Table 1) was as expected coronary artery disease (P < 0.01). Other statistically significant differences between the exposure groups included APACHE II score (14.9 vs 18.2, p = 0.03), initial white cell count (12.2 vs 16.8, p = 0.02), and 30 day survival (73% vs 37%, p = 0.02), which were all higher in the statin-user group. There were no significant differences between the two groups with *Candida *species obtained from blood cultures or duration or selected empiric antifungal therapy given to each group. (Table 2) The main statins observed to be used were atorvastatin (9/15, 60%), simvastatin (5/15, 33%) and pravastatin (1/15, 7%). In the matched conditional logistic bivariable analysis (table 3), statin exposure the only individual risk-factor associated with a statistically significant effect on mortality (p = 0.03, OR 0.09, 95%CI 0.11 - 0.746), while both higher APACHE II score and higher white blood cell count trended toward increased mortality (p = 0.08). In the multivariable conditional logistic regression analysis that included APACHE II score and statin therapy, statin exposure was associated with a statistically non-significant reduced mortality (OR 0.22, 95% CI 0.02-2.4, p = 0.21) compared to unexposed patients.

**Table 2:** *Candida *species and initial empiric antifungal medication received by exposure status

	Patients on Statins, N (%)	Patients Not on Statins, N (%)	p-value
***Candida Sp.***			

*Candida albicans*	6 (40%)	11 (37%)	0.3

*Candida glabrata*	4 (27%)	11 (37%)	0.5

*Candida parapsilosis*	3 (20%)	4 (13%)	0.57

*Candida tropicalis*	1 (7%)	4 (13%)	0.5

Mixed Candida*	2 (13%)	0	0.2

**Antifungal Therapy**			

Amphotericin B	1 (7%)	1 (3%)	1.0

echinocandin	7 (47%)	12 (40%)	0.75

fluconazole	7 (47%)	17 (57%)	0.54

* Mixed isolates were 1 *C. albicans *and *C. glabrata*, and 1 *C. albicans *and *C. parapsilosis*.

**Table 3:** Risk-factors for mortality*

Model	Exposure	Odds Ratio (95% CI)	p-value
1	Statin Exposure	0.09 (0.11-0.746)	0.03

2	Apache II	1.5 (0.95-2.4)	0.08

3	Male	1.8 (0.4-8.0)	0.46

4	WBC count	1.2 (0.98-1.4)	0.08

5	PTT	1.02 (0.96-1.085)	0.52

6	Renal insufficiency	0.30 (0.05, 1.7)	0.17

			

7	Statin	0.22 (0.02, 2.4)	0.21

	Apache II	1.4 (0.8, 2.3)	0.27

PTT = partial thromboplastin time; WBC = white blood cell

*For heart disease and heart failure in the single exposure models and white blood cell count in the multivariable model there were insufficient numbers in the strata to calculate a meaningful confidence interval.

## Discussion

This is the first study to evaluate the effects of statins on sepsis caused by candidemia in isolation from other causes. This study finds that there may be a clinical benefit of statin therapy in ICU patients with candidemia and an associated SIRS response. Within this small cohort, our data suggest statins given concurrently with antifungal therapy for candidemia were associated with a 91% reduction in mortality in the bivariable analysis and an attenuated 78% reduction in odds of mortality when controlling for severity of illness with APACHE II score. However the odds ratio (OR) can overestimate the relative risk (RR). In this case the RR = 0.42 for mortality in Table 1 (27%/63%) is much less protective than the OR would suggest. Candidemia is a marker of severe illness in the ICU and diagnosis is often delayed because of low blood culture yields [3,19]. With the overall candidemia related mortality over 30% in this setting, any extra benefit for drugs that modulate the immune system may be useful.

This finding is supported by in-vitro models suggesting that statins may interfere with ergesterol production in yeast [20]. The effect of statins on ergesterol production and cytokines may also explain why the statin group had a reduced inflammatory response compared to the controls in our study [10,15]. Macreadie et al. have demonstrated in-vitro that by supplementing *Candida albicans *with ergesterol in aerobic culture can overcome the inhibition caused by statins [13]. Importantly, Nash et al have demonstrated that the addition of pravastatin or lovastatin does not affect fluconazole activity [21].

There are now several larger retrospective studies showing the benefits of statins in bacterial sepsis. Initially, Liappis et al demonstrated that patients on statins had greater than 7 times greater chance of survival with sepsis [22]. Two recent prospective observational studies using large cohorts have also demonstrated similar findings. Almog et al showed in an ICU that only 2.4% of patients on a statin developed bacterial sepsis compared with 19% (p < 0.001) who were not on a statin [9]. Gupta et al also found that hemodialysis patients taking statins were also significantly less likely to be hospitalized for severe sepsis [5]. There have been several other retrospective and observational studies with similar findings with bacteremia [23-25].

The timing of initiation of statins with regards to the onset of sepsis is still being determined. This is because it takes several days for statin to achieve desirable concentrations [26]. Pre-admission use of a statin has shown relative risk reductions in large cohort studies evaluating community acquired pneumonia and ICU admission [27,28]. Thomsen et al showed that the use of preadmission statin up to 180 days prior to admission demonstrated a 25-30% mortality rate reduction at 90 days [27]. Also, Christensen et al showed about a 20% mortality rate reduction between statin users and non-users [28]. However Majumdar et al showed in a prospective cohort study for pneumonia that the benefit of statins disappeared after adjustment for confounders [6]. These confounders which Thomsen et al discuss include the "healthy-user" effects of patients being on statins with patients being better educated and socioeconomically better off than non-users [27]. We were unable to control for these "healthy-user" factors, but in an ICU setting may be less relevant. Based on our exclusion criteria to identify only SIRS induced by candidemia, every effort was made to find matching controls. Major differences were that statin-users were more likely to have heart failure, renal insufficiency, stroke, and peripheral vascular disease. Since our hypothesis was that statins would be protective and statins had higher comorbidity, we expect this to bias our findings to the null. Thus, if we were able to obtain perfect matching, we would have seen statins as being more protective.

The differences in APACHE II scores between the statin-users and unexposed groups, despite the statin-users having a higher Charlson comorbidity score could be either due to statin effect or how patients were matched. A prospective study by Novack et al randomly assigned ICU patients to receive a statin or not and evaluated their cytokine responses [12]. They found that statins significantly reduced TNF-α and IL-6 despite the statin goup having a higher APACHE II score [12]. Similarly, Schmidt et al showed that statin-users in the ICU with multi-organ dysfunction may have better survival with reduction in inflammatory markers [29].

We recognize our study limitations being the small sample size, with associated limited power, and patients were from a single center. In addition, we were not able to standardize the statins used given the retrospective nature of the study. There was exceptional difficulty in obtaining enough patients with candidemia without another cause of their SIRS and remaining on a statin throughout their antifungal therapy and then matching the cases and controls as closely as possible to our definitions. We suspect that most patients have their statins discontinued because of the lack of an intravenous formulation despite being able to deliver the drug down feeding tubes, although recent data suggest that this method may lead to elevated plasma levels of the statins [30].Our limitations presented are similar to other studies published on statins and bacteremic sepsis [27,28,31].

## Conclusions

This retrospective matched-cohort study suggests that there may be a survival benefit to remaining on a statin with a SIRS response from candidemia, but an overall statistically significant survival benefit was not observed when adjusted by APACHE II score. Further studies are warranted to define the optimal dose of statin and timing of therapy in a prospective manner. Additionally, larger studies are warranted to validate and further explore this association.

## Abbreviations

All abbreviations are defined within the manuscript.

## Competing interests

Dr Forrest has received research support and honoraria from Astellas Pharm, Inc. and Pfizer, Inc.

Dr Kopack has no conflicts of interest.

Dr Perencevich has received research support from Pfizer, Inc. and Merck, Inc.

## Authors' contributions

GF conceived the study, participated in the design and data collection and drafted the final manuscript. AK participated in the study design and performed the data collection and was involved in drafting the final manuscript. EP participated in the design, performed all the statistical analysis and in drafting and revising the final manuscript.

All authors read and approved the final manuscript.

## Pre-publication history

The pre-publication history for this paper can be accessed here:

http://www.biomedcentral.com/1471-2334/10/152/prepub
